# Magnetic Resonance Detection of CD34^+^ Cells from Umbilical Cord Blood Using a ^19^F Label

**DOI:** 10.1371/journal.pone.0138572

**Published:** 2015-09-22

**Authors:** Lucia E. Duinhouwer, Bernard J. M. van Rossum, Sandra T. van Tiel, Ramon M. van der Werf, Gabriela N. Doeswijk, Joost C. Haeck, Elwin W. J. C. Rombouts, Mariëtte N. D. ter Borg, Gyula Kotek, Eric Braakman, Jan J. Cornelissen, Monique R. Bernsen

**Affiliations:** 1 Department of Hematology, Erasmus University Medical Centre, Rotterdam, The Netherlands; 2 Department of Radiology, Erasmus University Medical Centre, Rotterdam, The Netherlands; 3 Department of Nuclear Medicine, Erasmus University Medical Centre, Rotterdam, The Netherlands; French Blood Institute, FRANCE

## Abstract

Impaired homing and delayed recovery upon hematopoietic stem cell transplantation (HSCT) with hematopoietic stem cells (HSC) derived from umbilical cord blood (UCB) is a major problem. Tracking transplanted cells *in vivo* will be helpful to detect impaired homing at an early stage and allows early interventions to improve engraftment and outcome after transplantation. In this study, we show sufficient intracellular labeling of UCB-derived CD34^+^ cells, with ^19^F-containing PLGA nanoparticles which were detectable with both flow cytometry and magnetic resonance spectroscopy (MRS). In addition, labeled CD34^+^ cells maintain their capacity to proliferate and differentiate, which is pivotal for successful engraftment after transplantation *in vivo*. These results set the stage for *in vivo* tracking experiments, through which the homing efficiency of transplanted cells can be studied.

## Introduction

Cell transplantation is an important therapeutic strategy for various malignant and non-malignant diseases. Migration of transplanted cells to their designated organs (‘homing’) is pivotal for treatment success. Information about transplanted cell localization can be of great value in the evaluation and development of stem cell-based therapies[1]. This information follows from magnetic resonance imaging (MRI) data, when cells of interest are labeled so that they can be discriminated from surrounding tissue. The stable nature of MRI cell labels facilitates longitudinal measurements, respecting the dynamic process of stem cell homing. Multiple studies have shown effective magnetic labeling and subsequent *in vivo* imaging in a variety of medical fields, including cardiovascular disease[2], neurodegenerative disease[3], neurological trauma[4], diabetes[5] and others. Using fluorine (^19^F) as a label has the advantage that ^19^F [6] has no detectable background *in vivo*[7]. Therefore, detection of ^19^F in cell labels is highly specific.

Labeling cells with ^19^F is mostly done using perfluorocarbons (PFCs), because PFCs are high in fluorine content[8]. Because of their insolubility in both lipophilic and hydrophilic solvents, PFCs need to be incorporated in emulsion droplets, nanoparticles or micelles before they can be used for cell labeling. Another reported ^19^F labeling strategy is to fluorinate sugars or peptides[9–11] but then the ^19^F content of the label is low compared to the ^19^F content of PFCs.

Transplantation of hematopoietic stem and progenitor cells from umbilical cord blood (UCB) is an example of the need for information about homing. UCB is an important alternative stem cell source for patients lacking a sibling or matched unrelated stem cell donor, because of its rapid availability and less stringent matching criteria[12]. However, adult patients who receive a UCB transplantation have a delayed neutrophil and platelet recovery time and a higher incidence of graft failure as compared to patients who receive CD34^+^ cells from adult donors [13, 14]. During the delayed recovery period, patients are at high risk for severe complications such as infections and bleeding, resulting in a high mortality rate. Several factors may contribute to the delayed hematopoietic recovery following UCB transplantation. Besides the relative higher immaturity of UCB-derived CD34^+^ cells as compared to adult bone marrow-derived CD34^+^ cells[15], delayed recovery may also be due to the relatively low number of CD34^+^ cells in UCB grafts[12, 16]. In addition, it is known that CD34^+^ cells derived from UCB do not home as efficiently to the bone marrow as their adult-donor-derived counterparts, due to a lack of binding of UCB-HSC to the P- and E-selectin adhesion molecules expressed by the recipients bone marrow endothelial cells[17]. Tracking stem cell homing after transplantation, as a means to study engraftment kinetics, will be helpful to detect impaired homing at an early stage after transplantation, allowing interventions to improve engraftment and outcome after transplantation.

In this preclinical study, the aim is to label umbilical cord blood (UCB)-derived CD34^+^ cells with fluorine (^19^F)-containing nanoparticles while maintaining cell viability and functionality. This will set the stage for further in vivo studies in order to track the homing of CD34^+^ cells upon hematopoietic stem cell transplantation.

## Material and Methods

### Synthesis and characterization of ^19^F-PLGA nanoparticles

Nanoparticles were produced as described [18]. Subsequently, the nanoparticles were resuspended in PBS (Invitrogen, the Netherlands) and stored at 4°C until use. The final concentration of ^19^F-PLGA nanoparticles was 76 mg/ml, as measured from a lyophilized sample.

Analysis of the ^19^F -PLGA nanoparticles using dynamic light scattering (Zetasizer Nano Series, Malvern Instruments, Worcestershire, UK) showed a mean particle diameter ± SD (n = 4) of 290 nm ± 56 nm. The mean polydispersity index ± SD was 0.17 ± 0.04, indicating particles were fairly homogenous in diameter.

### Umbilical cord blood processing and cell selection

Umbilical cord blood was collected in several hospitals using Stemcare®/CB collect blood bag system (Fresenius Kabi Norge AS) containing citrate phosphate dextrose (CPD) as an anticoagulant. The Medical Ethical Committee of the Erasmus University Medical Centre approved collection of the cord blood (MEC-2009-410) and written informed consent from the mother was obtained prior to donation. Within 48 hours after collection, mononuclear cells were isolated using ficoll (Lymphoprep™, Fresenius Kabi Norge AS). CD34^+^ cells were isolated with positive immunomagnetic selection using Magnetic Activated Cell Sorting (MACS) technology according instructions of the manufacturer (Miltenyi Biotech GmBH, Bergisch Gladbach, Germany).

### Labeling CD34^+^ cells with ^19^F -PLGA nanoparticles

Cells were resuspended at 200,000 cells/ml in serum-free Glycostem Basal Growth Medium (GBGM®, Glycostem Therapeutics, ‘s Hertogenbosch, The Netherlands) supplemented with thrombopoietin (TPO), stem cell factor (SCF) and Flt3 ligand (Flt3L) (Cellgenix, Freiburg, Germany) at 50 ng/ml each. Labeling of cells was performed at concentrations ranging from 5 to 40 μl/ml of ^19^F -PLGA nanoparticles. Standard concentration was 20 μl/ml. After addition of the label, cells were incubated in the dark at 37°C and 5.0% CO_2_ for 4 or 20 hours. Control cells were mock-labeled, i.e. treated identically until the end of the incubation time, but without the addition of nanoparticles. After incubation, cells were processed as required for further analysis.

### Flow cytometry

Labeling efficiency and median labeling intensity were determined using flow cytometry. Cells were stained with anti-CD45-APC-H7, anti-CD34-PE-Cy7 (both from BD Biosciences, San Jose, CA, USA) and diamidinophenylindole (DAPI) (Sigma-Aldrich, St Louis, MO, USA). Only viable (DAPI^-^) CD45^+^CD34^+^ cells were included in the analysis. The maximal fluorescence intensity of mock-labeled control cells in the FITC-channel was set as threshold for considering a cell labeled. Flow cytometric analysis was performed using a BD FACSCanto™ (BD Biosciences, San Jose, CA, USA) and data was analyzed using FlowJo software (Tree Star Inc, Ashland, OR, USA).

To assess ^19^F-PLGA labeling intensity in cells that divided 0–3 times, cells were labeled with 5μM CellTrace Violet (Molecular Probes, Eugene, Oregon, USA) upon ^19^F-PLGA labeling and subsequently cultured for 4 days in our culture medium as described above.

### Confocal microscopy

Cells were labeled at a ^19^F -PLGA nanoparticle concentration of 20 μl/ml with an incubation time of 4 hours. Labeled-cells were separated from free ^19^F -PLGA particles using ficoll separation. Washed labeled or mock-labeled control cells were transferred to microscopy slides by centrifugation. Subsequently, slides were air dried and mounted in Prolong® Gold Antifade Reagent with DAPI (Molecular Probes, Eugene, Oregon, USA). Cells were imaged using a Leica SP5 CLSM equipped with Ar-He/Ne lasers (Leica Microsystems, Wetzlar, Germany) and a Zeiss 63x Plan-Apochromat oil immersion objective (Carl Zeiss, Oberkochen, Germany). A 405 nm laser was used for DAPI excitation (with a 413–476 nm acousto-optical beam splitter (AOBS)), and a 488 nm laser was used for FITC excitation (with a 503–596 nm AOBS).

### Colony forming unit (CFU) assays

Washed labeled or mock-labeled cells were resuspended in methylcellulose containing medium (Methocult GF H84434, Stemcell Techonologies, Vancouver, BC, Canada) and seeded in triplicate at 500 cells per 35 mm dish. Dishes were incubated for 14 days in a humidified tray at 37°C and 5.0% CO2, after which two trained non-blinded observers enumerated the colonies. We accepted an interobserver variation of 10%. Three types of colonies were distinguished: burst forming unit-erythroid (BFU-E), colony forming unit-granulocyte/monocyte (CFU-GM) and colony forming unit-granulocyte/erythrocyte/monocyte/megakaryocyte (CFU-GEMM).

### Magnetic resonance spectroscopy

Cells were labeled at a concentration of 20 μl/ml for 20 hours and subsequently fixed in 4% formaldehyde solution for at least 15 minutes at room temperature, washed in PBS, and resuspended in agar solution (0.3%). An MR 901 Discovery 7T magnet (Agilent Technologies, Santa Clara, CA, USA) with a preclinical front-end (GE Healthcare, Little Chalfont, UK) was used for MRS acquisition. The system is equipped with a gradient set with a maximum gradient strength of 300 mT m-1, a rise-time of 600 T m-1 s-1 and an inner diameter of 310 mm. For transmission and reception, an in-house-built dual tuned ^1^H/^19^F single channel surface coil with a diameter of 2 cm was used. The ^19^F MRS spectrum was recorded using a EchoSCI sequence (TR/TE = 1250/15 ms, NEX = 128, FOV = 6 cm, slice thickness = 2,5 cm).

MRS processing was performed in SAGE 7.6.2 (GE Healthcare, Little Chalfont, UK) on the MR 901 Discovery system. For processing of the data, time domain signals were apodized with a 17.6 Hz line broadening function, after which the signal was zerofilled to 4096 points. Subsequently the time domain signal was Fourier transformed and the resulting spectrum was properly phased to show an absorption mode resonance line. For signal intensity the maximum intensity of the resonance line was determined, and noise was estimated from the standard deviation of the signal intensity of the baseline. ^19^F in the sample was quantified by reference to a standard curve, which was obtained by measuring a dilution series of PFCE with known ^19^F content.

### Statistics

Unpaired two-tailed t-tests were performed to test the difference in median labeling intensity between different incubation times and to test the difference in number of colonies between labeled and mock-labeled CD34^+^ cells for both incubation times in CFU experiments. Differences were considered to be statistically significant if p < 0.05. Statistical tests were performed in Excel (Microsoft Corporation, Redmond, WA, USA).

## Results

### CD34^+^ cells are efficiently labeled with ^19^F -PLGA nanoparticles

**Fig 1:**
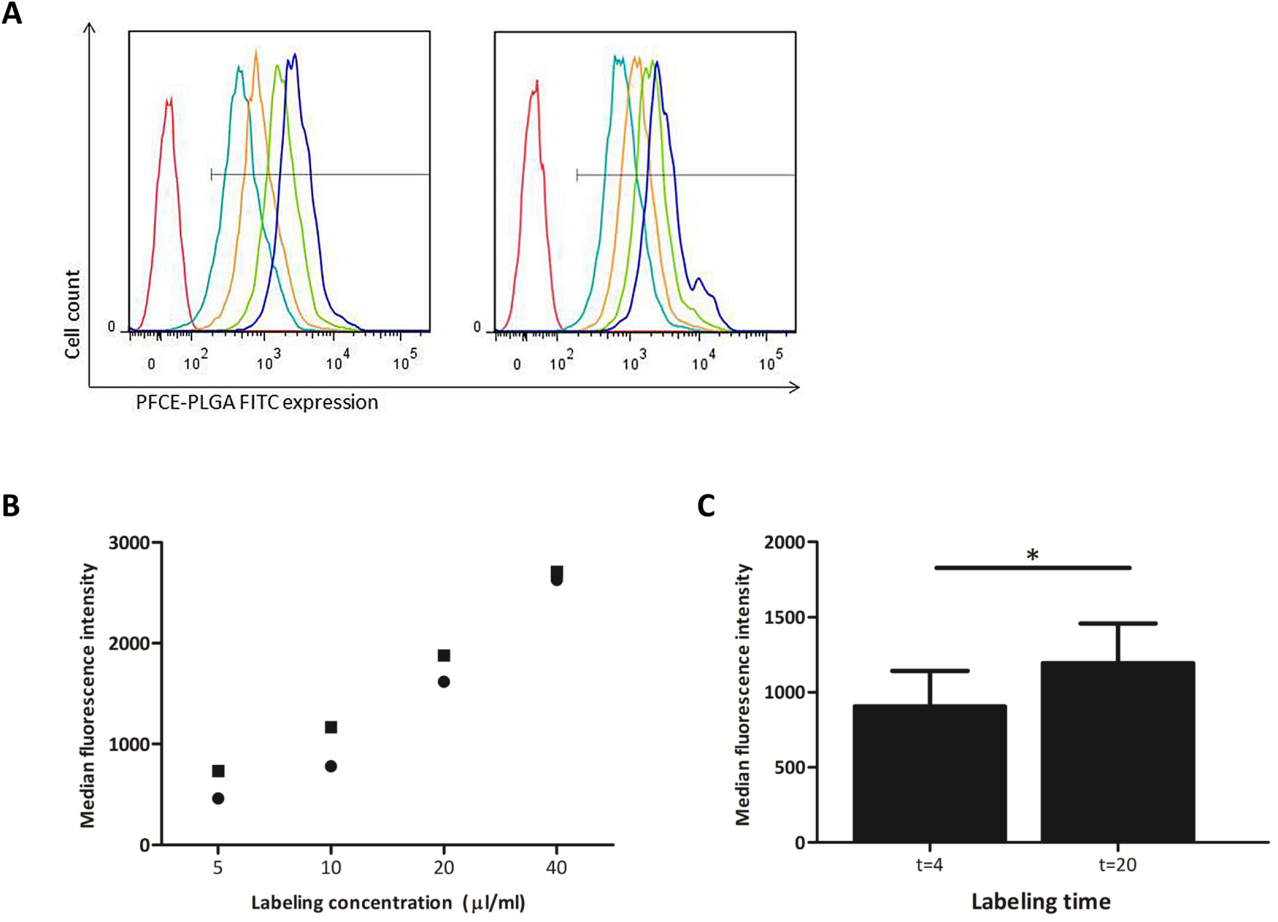
CD34^+^ cells can be labeled efficiently with ^19^F -PLGA nanoparticles with the intensity increasing with longer incubation time and higher labeling concentration. (A) Fluorescence histograms of cells labeled with 0 (red), 5 (turquoise), 10 (orange), 20 (green) and 40 (blue) μl/ml nanoparticles at incubation times of 4 (left panel) or 20 (right panel) hours. Horizontal axes show the intensity of the FITC signal, representing the ^19^F -PLGA nanoparticles. (B) Median fluorescence intensity per labeling concentration after 4 (circle) and 20 (square) hours of labeling. Figs 1A and 1B show a representative experiment out of 2 experiments. (C) Median fluorescence intensity of cells labeled with 20 μl/ml ^19^F -PLGA nanoparticles for 4 and 20 hours (n = 5). * = p<0.05.

Cells were labeled with 5, 10, 20 and 40 μl/ml ^19^F -PLGA nanoparticles for 4 or 20 hours respectively, in order to address whether CD34^+^ cells could be labeled using ^19^F -PLGA nanoparticles and which labeling time and concentration would be most optimal. In all conditions tested, incubation of CD34^+^ cells with ^19^F -PLGA nanoparticles resulted in labeling of nearly all cells. The lowest percentage of labeled cells (94.5%) was observed following incubation with a ^19^F -PLGA nanoparticle concentration of 5 μl/ml for 4 hours; all other conditions resulted in > 99% labeled cells (Table 1). However, the median fluorescence intensity of labeled cells varied across labeling conditions (Table 1, Fig 1A). Higher ^19^F -PLGA nanoparticle concentrations were associated with higher median fluorescence intensity of viable CD45^+^CD34^+^ cells. At each labeling concentration, a longer incubation time was associated with higher median fluorescence intensity of CD34^+^ cells (Fig 1B). However, the increase in median fluorescence caused by longer incubation time consistently decreased with increasing labeling concentration. An incubation time of 20 hours led to a statistically significantly higher median labeling intensity than 4 hours of labeling at 20 μl/ml (Fig 1C, p<0.05). In summary, we can discriminate labeled CD34^+^ cells from mock-labeled control cells using FACS in each of the conditions tested, with the labeling intensity increasing with longer incubation time and higher labeling concentration. We chose a labeling time of 20 hours with a concentration of 20 μl/ml for further experiments.

**Table 1:** Labeling efficiency of CD34^+^ cells in relation to ^19^F -PLGA NP concentration and incubation time.

Labeling concentration (μl/ml)	Percentage of cells labeled (%)		Median fluorescence intensity	
	*4 hours incubation*	*20 hours incubation*	*4 hours incubation*	*20 hours incubation*
5	94.5	99.2	461	733
10	99.1	99.9	781	1169
20	99.7	99.9	1618	1879
40	99.7	99.9	2628	2709

PLGA: poly(lactic-co-glycolic acid); NP: nanoparticle

### Detection of ^19^F-PLGA labeled CD34^+^ cells by magnetic resonance spectroscopy

**Fig 2:**
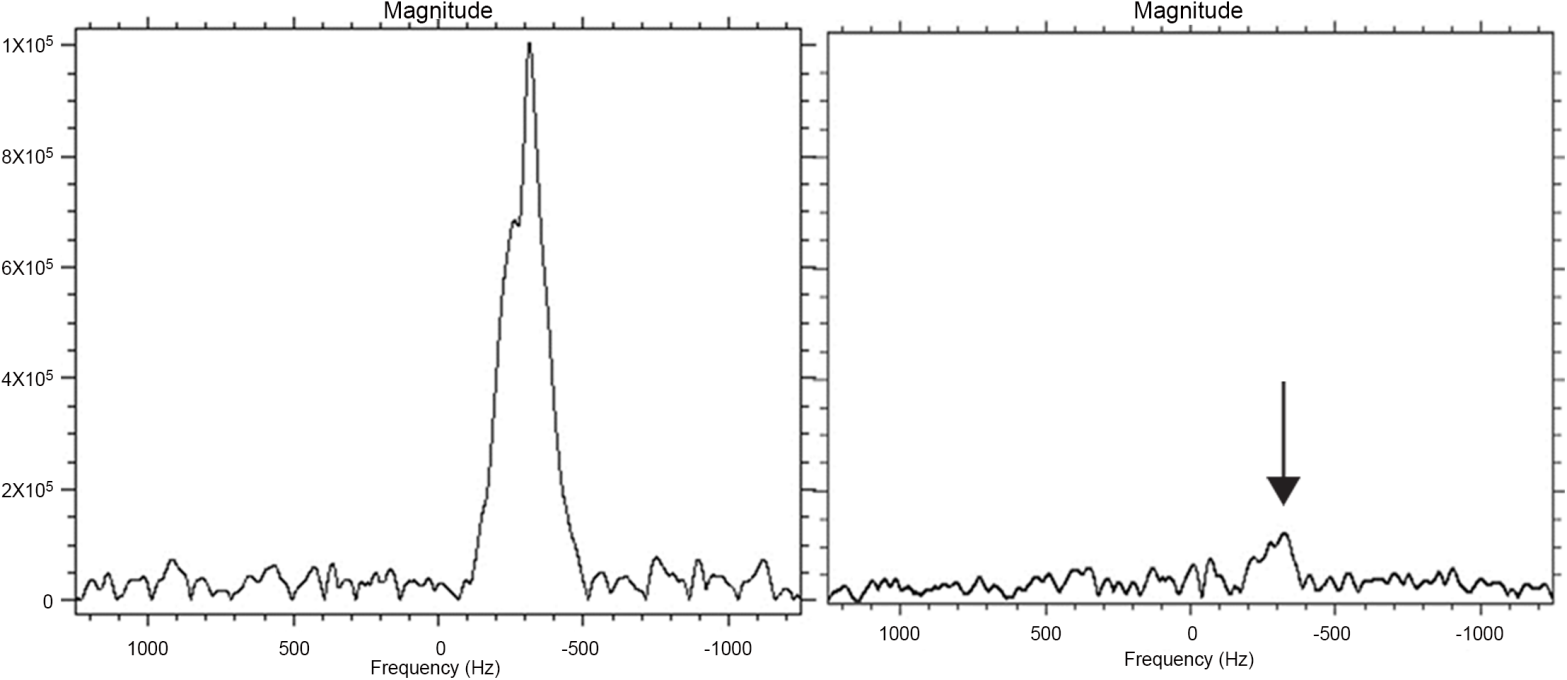
Detection of labeled CD34^+^ cells by magnetic resonance spectroscopy and imaging. Left and right panel show the ^19^F MRS spectrum of 2 agar gel phantoms containing 10^5^ and 10^4^ labeled CD34^+^ cells in 150 μl respectively. Shown is the ^19^F resonance line, the horizontal axes shows the frequency offset from the transmitter. Here the transmitter frequency has been set to the resonance frequency of the ^19^F in PFCE. Labeled cells were labeled with 20 μl/ml ^19^F -PLGA nanoparticles with an incubation time of 20 hours.

We recorded a ^19^F MRS spectrum of 2 agar gel phantoms containing 10^5^ and 10^4^ labeled CD34^+^ cells in 150 μl respectively. The respective signal to noise ratio (SNR) was 58,6 for the sample containing 10^5^ labeled CD34^+^ cells and 5,3 for the sample containing 10^4^ labeled CD34^+^ cells (Fig 2). Data acquisition took 65 minutes to recover sufficient SNR in the sample containing 10^4^ cells. 5,06x10^19 19^F spins were measured in the sample with 10^5^ labeled CD34^+^ cells and 6,65x10^18 19^F spins in the sample with 10^4^ labeled CD34^+^ cells.

### Uptake of the label is an active process and results in intracellular accumulation of the label

**Fig 3:**
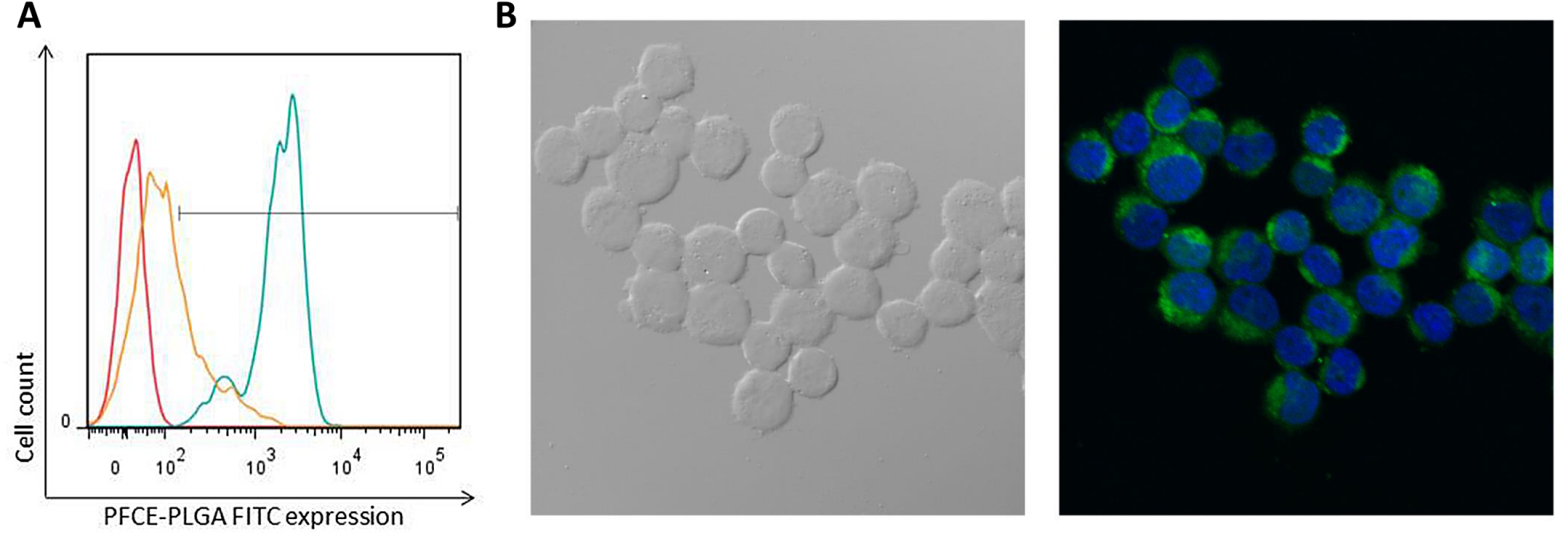
Uptake of the label is an active process and results in intracellular accumulation of the label. (A) Fluorescence histogram for mock-labeled control cells (red) and cells labeled 20 hours at 37°C (turquoise) or 4°C (orange). Horizontal axes show the intensity of the FITC signal, representing the ^19^F -PLGA nanoparticles. (B) Differential Interference Contrast image (left) and fluorescent image (right) of CD34^+^ cells labeled with ^19^F -PLGA nanoparticles, showing the blue DAPI-signal (nucleus of the cell) and the green FITC-signal (^19^F -PLGA nanoparticles).

To discriminate between active uptake of the label and binding of the label to the membrane, we performed labeling with 20 μl/ml ^19^F -PLGA nanoparticles at both 4°C and 37°C. We observed a decrease in frequency of labeled cells from 99.9% at (37°C) to 28.2% (at 4°C). In addition, the median fluorescence intensity decreased from 2061 after labeling at 37°C to 82.2 after labeling at 4°C (Table 2 and Fig 3A). This indicates that CD34^+^ cells actively incorporate the ^19^F -PLGA nanoparticles. To further investigate the intracellular accumulation of the label, we performed confocal microscopy of ^19^F -PLGA-labeled CD34^+^ cells. Fig 3B clearly shows FITC-positive ^19^F -PLGA nanoparticles within the cytoplasm of the labeled CD34^+^ cells. Mock-labeled control cells showed no FITC-signal (image not shown). Combined, these data show active uptake of the label by the CD34^+^ cells, leading to intracellular accumulation of the label.

**Table 2:** Effect of incubation temperature on uptake of ^19^F -PLGA NPs by CD34^+^ cells.

Incubation temperature	Percentage of cells labeled (%)	Median fluorescence intensity
37°C	99.9%	2061
4°C	28.2%	82.2

PLGA: poly(lactic-co-glycolic acid); NPs: nanoparticles

### ^19^F-PLGA labeling does not affect the relative proportion of committed hematopoietic progenitors and cell viability

**Fig 4:**
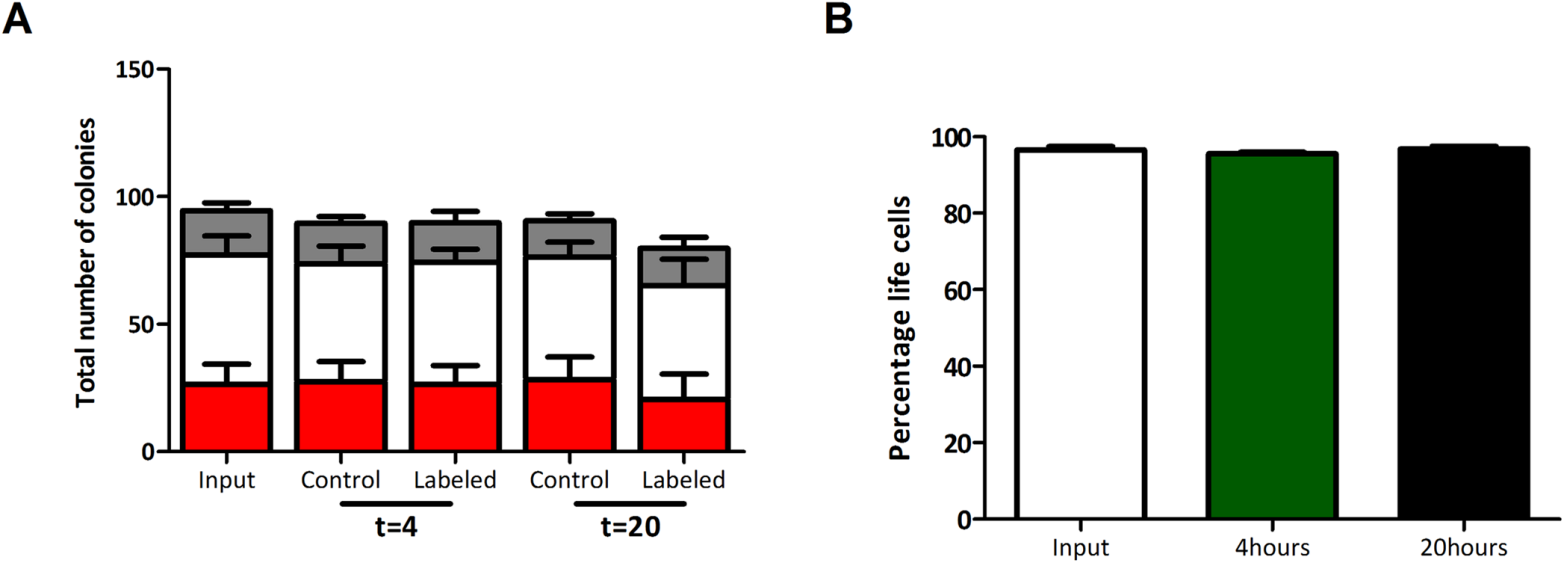
Labeling with ^19^F-PLGA does not affect the relative proportion of committed hematopoietic progenitors and cell viability. **(A)** Total number of colonies per 500 CD34^+^ cells for BFU-E (red), CFU-GM (white) and CFU-GEMM (gray). (B) Percentage of life cells at input (white), and after 4 (green) and 20 (black) hours of labeling with 20 μl/ml ^19^F-PLGA (n = 5).

To test the ability of the labeled cells to proliferate and differentiate, colony forming unit (CFU) assays were performed. ^19^F -PLGA labeled and mock-labeled CD34^+^ cells were plated on the basis of baseline cell counts (counted prior to labeling) and after 14 days of culture, three different colony types were scored by 2 trained observers. Relevant to the interpretation of CFU results are the total number of colonies and the relative distribution of colony types, indicating the relative proportion of distinct committed hematopoietic progenitors, compared between labeled and mock-labeled conditions. With an incubation time of 4 hours, the average total number of colonies and the relative distribution of the colony subtypes are similar among the labeled and control conditions (Fig 4A, p = 0.96 for the total number of colonies and p = 0.31, p = 0.53 and p = 0.83 for the frequency of BFU-E, CFU-GM and CFU-GEMM respectively). With an incubation time of 20 hours, the total number of colonies in the labeled condition is lower than in the mock-labeled control (90.6 versus 79.7 colonies per 500 CD34^+^ cells in control and labeled cells respectively, p = 0.04), although no significant decrease is observed in any of the colony types. Irrespective of this small decrease in total colony number after 20 hours of labeling, these results show that both after 4 and 20 hours of labeling, cells are still capable of proliferation and differentiation and the relative proportions of committed hematopoietic progenitors are similar.

To study whether ^19^F-PLGA labeling affects cell viability, flow cytometric analysis was performed using diamidinophenylindole (DAPI). The percentage life CD34^+^ cells prior to labeling and after labeling with ^19^F -PLGA for 4 and 20 hours were similar (Fig 4B), indicating no toxic effect of the internalization of the ^19^F -PLGA nanoparticles.

### ^19^F-PLGA labeled cells remain detectable over time and upon cell division, although label intensity decreases

**Fig 5:**
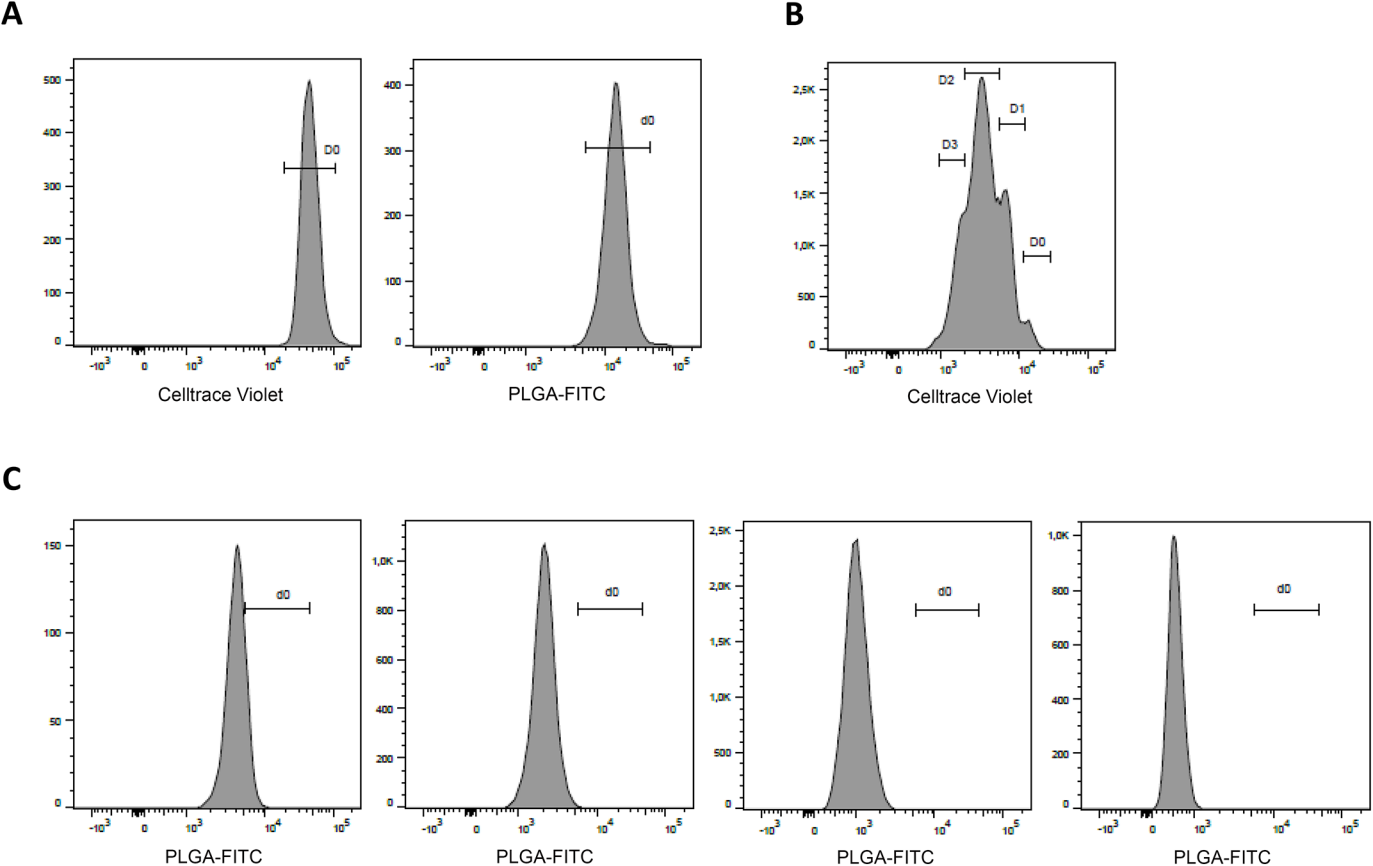
^19^F-PLGA labeled cells remain detectable over time and upon cell division, although label intensity decreases. Fluorescence histograms for CellTrace and ^19^F -PLGA-labeled CD34^+^ cells **(**A) Population shown is labeled cells at day 0. Horizontal axes show the intensity of the CellTrace Violet signal (left panel) and of the FITC signal, representing the ^19^F -PLGA nanoparticles (right panel). (B) Population shown is labeled cells after 4 days of culture. The horizontal axes show the intensity of the CellTrace Violet signal, indicating the number of cell divisions. (C) Populations shown are labeled cells after 4 days of culture who had undergone 0, 1, 2 or 3 cell division respectively (depicted in the panels from left to right). Horizontal axes show the intensity of the FITC signal, representing the ^19^F -PLGA nanoparticles.

To assess the stability of the label in non-dividing cells and loss of label intensity upon cell division, we labeled ^19^F -PLGA-labeled CD34^+^ cells with the cell division tracker CellTrace and evaluated the fluorescence intensity upon 4 days of culture. At day 0, all cells showed a high intensity of both the CellTrace label and the ^19^F -PLGA label (Fig 5A). After 4 days of culture, cells had divided 0, 1, 2 or 3 times respectively (Fig 5B). We observed a decrease in fluorescence intensity in cells that had not divided, compared to the day 0 population (Fig 5C, left panel), indicating some leakage of the label in time. However, labeled cells were still easily detectable using flow cytometry. In addition, upon every cell division we observed a halving of the fluorescence intensity, indicating an equal distribution of the ^19^F -PLGA nanoparticles over both daughter cells upon cell division. Despite the decrease in label intensity, labeled cells were still detectable by flow cytometry upon 3 cell divisions (Fig 5C, right panel).

## Discussion

This study shows the feasibility of labeling CD34^+^ cells with ^19^F-containing PLGA nanoparticles. In addition, labeled CD34^+^ cells maintain viable and retain their capacity to proliferate and differentiate, which is pivotal for successful engraftment after transplantation *in vivo*. In the future, this technique can be used to monitor homing evaluating efficacy of hematopoietic stem cells transplantation and the information obtained may have implications to further improve treatment outcome.

Several conditions must be met in order to consider cell labeling and subsequent MR detection as feasible. Firstly, MR detection of the labeled cells should be feasible *in vivo*. MR-based imaging has advantages regarding its high spatial resolution, the absence of ionizing radiation and the ability to provide anatomical information[1]. However, as compared to nuclear imaging of isotopes, MRI may be a less sensitive technique, with a higher detection limit in a reasonable measurement time. Higher MR detection sensitivity may provide more detailed information about the distribution of the labeled cells after transplantation. Since MRS has a higher sensitivity than MRI, we decided to first perform MRS experiments in order to create a starting point from which our MRS results can serve for increasing signal and cell detection sensitivity. This may be achieved by optimizing the ^19^F content of the nanoparticles, increasing the cellular uptake of the label and refining MR hardware and acquisition[19]. A second prerequisite involves preservation of viability and functionality of the cells during the labeling process. We observed comparable percentage of life cells and total colony numbers after an incubation period of 4 and 20 hours compared to control samples. In addition, all different types of colonies were formed by the labeled cells, in similar frequencies as by non-labeled cells. Thirdly, stable cell-label association is pivotal for successful MR detection of labeled cells. The stability of the cell-label association is partly determined by the localization of the label. Both surface labels and intracellular labels may be applied, but intracellular labels may be preferred due to a lower risk of detachment. Our data indicates that cellular label uptake is an active, energy-dependent process, resulting in a stable intracellular localization of the label. This finding is consistent with the results of previous studies, which identified endocytosis as the cellular mechanism responsible for uptake of PLGA NPs in other cell types[20–22]. Future research should address the question whether such intracellular labels are useful to investigate homing of transplanted CD34^+^ cells in the first 24 hours after transplantation. We observed decrease in label intensity over time and dilution of the signal upon cell division. This dilution of the signal may have consequences in imaging sensitivity, provided the 19F concentration per imaging voxel is reduced by migration of cells out of the area of interest. However, when cells do not migrate upon division dilution through cell division would not affect detection sensitivity since for 19F MRI/MRS the total amount of 19F in a voxel determines detection sensitivity and not how it is spread within a voxel. This was already shown in previous studies using gadolinium-DTPA containing liposomes to label mesenchymal stem cells. Guenoun et al. showed a stable amount of label in all cells over time, even though the amount per cell decreased as a result of mitosis[23]. In our studies, the voxel dimensions were dictated by the area of interest and a similar approach could be followed for clinical applications. Lastly, in order to develop this technique further also for clinical applications, it is crucial that the label is biocompatible. All components of our nanoparticles are biocompatible and are already used in other applications in humans. PLGA polymer is approved by the FDA and European Medicine Agency for use in humans as a drug delivery system[24, 25]. Because of their susceptibility to hydrolysis and subsequent clearance by the Krebs cycle[26], PLGA polymers have very minimal systemic toxicity[27]. PFC emulsion droplets are cleared by macrophages of the reticuloendothelial system and eliminated from the body by exhalation[28]. Possible adverse effects are caused by stimulation of the macrophages and are dose-dependent. Therefore, they may not apply to cell tracking studies using low doses of PFC[8]. Finally, PVA is used as an emulsifier in the production of PFCE-PLGA nanoparticles. Some PVA remains despite extensive washing of the nanoparticles[29]. PVA is biocompatible and applied in humans through oral administration[30] or implantation[31].

Helfer et al. [32]labeled CD34^+^ cells from adult bone marrow using a ^19^F label in emulsion droplets. Similar to our results, they found an increase in labeling intensity and frequency of labeled cells with increasing labeling concentration. However, we found no evidence of a detrimental effect of labeling on viability or functionality after 4 hours of labeling, whereas Helfer et al. found a slight decrease in viability. Both studies measured comparable ^19^F payloads. In addition, Partlow et al. showed internalization of ^19^F label in emulsion droplets in UCB mononuclear cells that were grown towards endothelial cells[6], which are different from the CD34^+^ cells we used in our study. The cells remained functional *in vivo* as well. We preferred to incorporate ^19^F in PLGA nanoparticles, because these are more stable than emulsion droplets, easier to store and the association with fluorescent dyes is more stable.

In a recent study, Ahrens et al. efficiently labeled human dendritic cells with a clinical grade PFC agent without changes in viability or phenotype. In this phase 1 study, patients suffering from stage IV colorectal cancer subsequently received intradermal administration of 1x10^6^ or 1x10^7^ labeled dendritic cells and underwent a MRI scan at 2 and 24 hours after administration. In the patients that received 1x10^6^ dendritic cells, no ^19^F signal was observed. However, 1x10^7^ administered dendritic cells could be detected by MRI at both the 2 and 24 hour time point, although the number of dendritic cells decreased to approximately half of the original values at 24 hours, due to either cell efflux, cell migration or cell death[33]. Although dendritic cells are different from CD34^+^ cells as used in our experiments and the required number of injected cells is high, these results are very promising first steps in *in vivo* tracking of labeled human cells.

In conclusion, CD34^+^ cells can be labeled efficiently with PFCE-PLGA NPs without affecting cell functionality or viability. Labeled cells can be detected using MRS on a 7T MRI scanner. These results set the stage for *in vivo* tracking experiments, through which homing efficiency of transplanted cells can be studied.
